# Coronoidaufbau mittels Beckenkammspan über einen medialen Zugang zur Therapie von chronischen Coronoiddefekten mit posteromedialer Rotationsinstabilität

**DOI:** 10.1007/s00064-022-00783-6

**Published:** 2022-09-08

**Authors:** M. M. Schneider, F. Zimmermann, B. Hollinger, A. Zimmerer, K. J. Burkhart

**Affiliations:** 1 Arcus Sportklinik, Rastatter Str. 17–19, 75179 Pforzheim, Deutschland; 2 Universität Witten/Herdecke, Ostmerheimer Str. 200, 51109 Köln, Deutschland; 3 Abteilung Sportorthopädie, Orthopädische Klinik Markgröningen, Markgröningen, Deutschland; 4 Universität zu Köln, Köln, Deutschland

**Keywords:** Anteromediale Facette, PMRI, Coronoidrekonstruktion, Instabilität, Coronoidplastik, Anteromedial facet, Posteromedial rotatory instability, PRMI, Varus posteromedial rotatory instability, Coronoid deficiency

## Abstract

**Operationsziel:**

Ziel ist die Wiederherstellung der ulnohumeralen Gelenkkongruenz mit Neutralisierung der posteromedialen Instabilität (PMRI) und der damit einhergehenden chronischen Fehlstellung des Ellenbogens.

**Indikationen:**

Neben akuten Defekten, auf die im Artikel nicht näher eingegangen werden soll, besteht die Indikation zum Coronoidaufbau bei chronischen Defekten der anteromedialen Facette des Coronoids mit PMRI.

**Kontraindikationen:**

Gegen die Durchführung der Operation sprechen fortgeschrittene Knorpelschäden des Ellenbogens sowie allgemeine Kontraindikationen (akuter Infekt, Schwangerschaft, fehlende Operabilität des Patienten etc.)

**Operationstechnik:**

Über einen medialen Zugang wird zunächst das Coronoidbett präpariert. Im Anschluss wird ein autologer Beckenkammspan auf den Defekt aufgebracht, an die Gelenkfläche anpasst und mittels Schrauben- oder Plattenosteosynthese dauerhaft fixiert. Additiv wird eine Rekonstruktion des anterioren Bündels des medialen Kollateralbandes durchgeführt und die Kapsel bzw. Flexoren werden zum Ende der Operation verschlossen respektive rekonstruiert.

**Weiterbehandlung:**

Postoperativ wird eine Bewegungsorthese angepasst, die für 6 Wochen getragen werden sollte. Der Ellenbogen kann unmittelbar nach Operation schmerzadaptiert frei funktionell beübt werden. Die Therapie wird durch einen CPM-Ellenbogenstuhl unterstützt.

**Ergebnisse:**

Zwischen 04/2015 und 11/2017 wurden 10 Patienten mit chronischen Coronoiddefekten mit dieser Technik operiert. Acht der 10 Patienten standen 86 Wochen postoperativ für ein Follow-up zur Verfügung. Die durchschnittlich 41,4 Jahre alten Patienten wiesen postoperativ alle einen gesteigerten Bewegungsumfang, regelrechte radiologische Stellungskontrollen ohne Subluxationen und Verbesserungen in Funktionsscores auf. Ein Patient wurde ausgeschlossen, weil eine Konversion in eine Ellenbogenprothese notwendig wurde. Mit der beschriebenen Technik ließ sich eine objektive wie subjektive Verbesserung der chronischen PMRI erreichen.

## Abstract (en)

**Objective:**

Neutralizing a posteromedial rotatory instability (PMRI) caused by coronoid deficiency by restoration of the humeroulnar joint surface with an autologous iliac crest bone graft.

**Indications:**

Surgery is indicated in patients with chronic deficiency of the anteromedial facet of the coronoid with subsequent PMRI.

**Contraindications:**

Coronoid reconstruction is not recommended in patients with advanced osteoarthritis of the elbow caused by subluxation of the humeroulnar joint. General contraindications like acute infection, pregnancy and lack of operability should also be taken into account.

**Surgical technique:**

First, a medial approach is established and the base of the coronoid is prepared. Afterwards an autologous iliac crest bone graft is placed onto the defect and secured by screws or a plate. In addition, a reconstruction of the anterior bundle of the medial collateral ligament with an autologous tendon graft is performed.

**Postoperative management:**

An elbow orthesis is worn for 6 weeks after surgery to avoid valgus or varus stress. There is no restriction in range of motion. A continuous passive motion elbow chair supports the patient in regaining elbow mobility.

**Results:**

Between 2015 and 2017, we treated 10 patients suffering from chronic coronoid defects with coronoid reconstruction. Eight of the patients were available for follow-up 86 weeks after surgery. The mean age was 41.4 years. In all patients, elbow range of motion and patient-related outcome measures were improved after surgery. Plain radiographs illustrated correct centering of the elbow joint. One patient had to undergo elbow arthroplasty and was excluded. Coronoid reconstruction with an autologous iliac crest bone graft restored humeroulnar joint congruency and improved satisfaction in patients suffering from chronic coronoid deficiency.

## Vorbemerkungen

**Figure 1:**
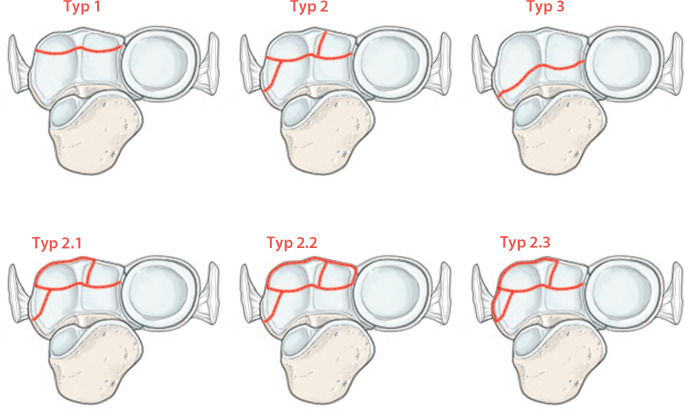

Der Ellenbogen hat in den letzten Jahrzenten mehr und mehr an Interesse gewonnen [1–4]. Das Coronoid dient der passiven Stabilisierung [5–7], stellt die entscheidende ulnare Barriere gegen eine posteriore Luxation des Ellenbogens dar [8, 9] und wird nicht umsonst als „Keystone“ des Ellenbogens bezeichnet [10, 11]. Isolierte Frakturen des Coronoids sind selten und treten mit einer Inzidenz von ca. 2,1 bis 7,5 pro 100.000 auf [12, 13]. Dabei ist zu beachten, dass die Rate an knöchernen oder ligamentären Begleitverletzungen bei Coronoidfrakturen mit 73 % enorm hoch ist [12]. In den meisten Fällen werden Coronoidfrakturen als Teil komplexer Ellenbogenluxationsfrakturen beobachtet [14, 15]. Dem Coronoid wird in der Fraktursituation oftmals nicht die ausreichende Beachtung geschenkt bzw. das Ausmaß der Fraktur unterschätzt [16, 17], so dass es immer wieder zu chronischen posttraumatischen Defektsituationen mit Entstehung einer posteromedialen Rotationsinstabilität (PMRI) durch insuffiziente Frakturversorgung kommen kann [5, 6, 15]. Eine PMRI entsteht nicht nur bei basisnahen Frakturen des Coronoids, sondern auch bei Beteiligung der anteromedialen Facette [5, 6, 18, 19]. Aufgrund der hohen klinischen Relevanz der PMRI entwickelte O’Driscoll eine eigene Frakturklassifikation, welche die ursprüngliche Klassifikation von Regan und Morrey [20] um die Frakturen der anteromedialen Facette erweiterte (Abb. 1; [21]). Ursache für die akute PMRI des Ellenbogens ist v. a. die Coronoidfraktur des Typs 2. Aufgrund des Traumamechanismus [10, 22] ist die PMRI mit einer Ruptur des lateralen Kollateralbandkomplexes (LCL) sowie des posterioren Bündels des medialen Kollateralbandes (PMCL) assoziiert. Das anteriore Bündel des MCL (AMCL) kann bei der Verletzung betroffen sein, muss es allerdings nicht zwingend. Durch die fehlende knöcherne Abstützung durch den Defekt der anteromedialen Facette kommt es zu einem erhöhten Druck zwischen der medialen Trochlea und dem frakturierten Coronoid [21], was unbehandelt zu einer zeitnahen Entwicklung einer Ellenbogenarthrose führen kann [8, 21, 23].

In der chronischen Defektsituation ist die schnellstmögliche Rezentrierung des Gelenks von essenzieller Bedeutung. Es existieren verschiedene Techniken, die fehlenden anteromedialen Anteile der Incisura trochlearis zu rekonstruieren und damit die Kongruenz der Gelenkpartner wiederherzustellen [17, 24–29]. Vergleichende Studien zu den verschiedenen Techniken existieren nicht. Wir stellen im Folgenden den Aufbau des Coronoids mittels autologen Beckenkammspans und additiver medialer Bandplastik mittels Gracilissehne (wahlweise auch Palmaris-longus-Sehne) über einen medialen Zugang vor. Der mediale Zugang erlaubt dabei eine gute Sicht auf die Defektsituation des Coronoids und unterstützt die stufenlose Wiederherstellung der Gelenklinie. Im gleichen Eingriff oder frühsekundär (nach ca. 6 Wochen) ist meist eine Plastik des lateralen ulnaren Kollateralbandes (LUCL) erforderlich [30].

### Anatomie

**Figure 2:**
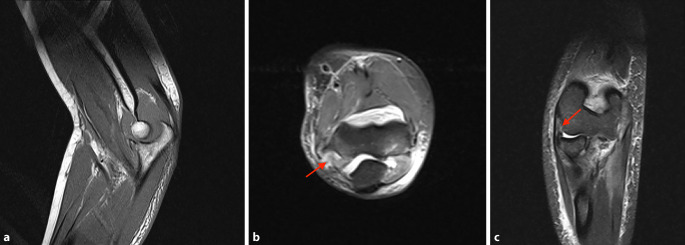

Die Gelenkpartner im Humeroulnargelenk werden gebildet durch die Trochlea humeri und die sie C‑förmig umfassende Incisura trochlearis. Das Coronoid stellt den ventralen Anteil der Incisura trochlearis dar und wird unterteilt in den Apex, die Basis, das Tuberculum subliminus und die anteromediale Facette (Abb. 1). Am Tuberculum subliminus setzt das anteromediale Bündel (AML) des medialen Kollateralbandes (MCL) an [31–33], das den primären Stabilisator gegenüber Valgusstress darstellt. Das mediale Kollateralband zählt zu passiven weichteiligen Stabilisatoren. Es besteht aus 3 Teilen: dem genannten anterioren Bündel (AML), einem posterioren Bündel (PML) und transversen Fasern [33]. Ihren gemeinsamen Ursprung haben das AML und PML am Epicondylus ulnaris des Humerus. Im Gegensatz zum AML inseriert das PML am Olekranon [31, 32]. Häufig ist aufgrund des Traumamechanismus dann auch das laterale ulnare Kollateralband (LUCL), welches am Epicondylus radiale entspringt und in das Lig. anulare radii einstrahlt [33], rupturiert (Abb. 2).

### Klinische Untersuchung

Die Anamnese sollte immer die Frage nach einem Trauma beinhalten. Die standardisierte Untersuchung beginnt dann mit der Inspektion (Schwellung/Hautverletzungen/Hämatome) und Überprüfung der peripheren Durchblutung, Motorik und Sensibilität. Anschließend werden aktiver und passiver Bewegungsumfang erfasst. Nicht selten werden dabei durch die Subluxationsstellung Bewegungseinschränkungen im Sinne einer Ellenbogensteife beobachtet. Abhängig vom Zeitpunkt der Untersuchung (akut vs. chronisch) kann eine Bewegung unter forcierter Muskelanspannung ein vernehmbares Krepitieren auslösen, was auf das Vorliegen von Knorpelschäden hindeuten kann. Nach Überprüfung möglicher Schmerzpunkte (Epikondylen, Soft Spot, MCL-Verlauf) wird die Stabilität des N. ulnaris getestet. Abschließend erfolgt die Stabilitätsprüfung der Bandkomplexe. Neben der gängigen Prüfung der posterolateralen Rotationsinstabilität (PLRI) z. B. mittels Pinzettengriffs oder lateralen Pivot-Shift-Tests bietet sich medial nicht nur der Valgusstresstest bzw. das Milking-Manöver (Prüfung des AMCL) an, sondern auch der Varusstresstest für die Detektion der PMRI [7]. Dabei führt der Patient in einer 90° Abduktionsstellung im Glenohumeralgelenk langsame Extensions- und Flexionsbewegungen im Ellenbogen durch. Bei vorliegender PMRI kommt es durch die Schwerkraft entweder zu Schmerzen, einem Instabilitätsgefühl oder Krepitationen im Ellenbogen. Ein exakter Nachweis oder eine Provokation der Subluxation – mit Ausnahme von ausgeprägten Fällen – erscheint klinisch allerdings schwierig, sodass die Diagnose der PMRI tendenziell eher über die radiologische Bildgebung gestellt wird.

### Radiologische Untersuchung

**Figure 3:**
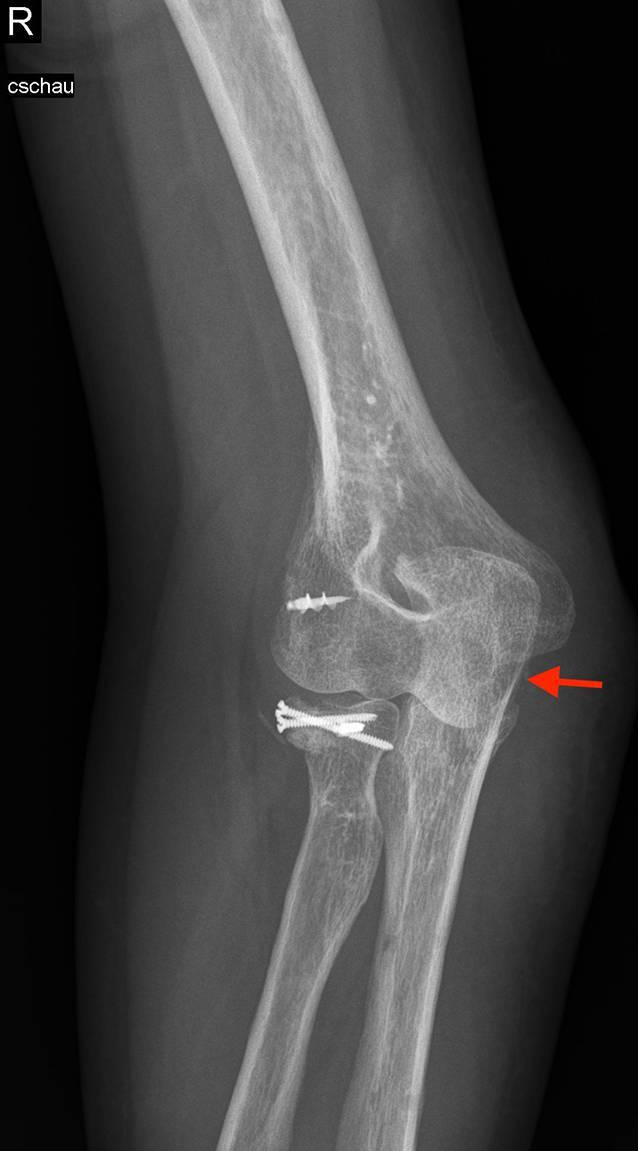

Die radiologische Basisdiagnostik mit einer Röntgenbildgebung in 2 Ebenen kann sowohl in der akuten als auch chronischen Situation wichtige Hinweise auf das Verletzungsmuster liefern. Nur in der anteroposterioren Aufnahme ist die Subluxationsstellung deutlich erkennbar (Abb. 3). In der lateralen Aufnahme zentriert das Gelenk durch die durchgeführte Flexion, so dass keine Fehlstellung auffällt. Bei röntgenologischem Verdacht auf eine vorliegende chronische Coronoiddefektsituation ist im Anschluss die Durchführung einer Schnittbildgebung einzuleiten. In der Magnetresonanztomographie (MRT) oder Computertomographie (CT) lässt sich dann meist eine mehr oder weniger ausgeprägte posteromediale Subluxationsstellung erkennen. Darüber hinaus können in der MRT die Kollateralbänder und grob auch die Knorpelsituation bzw. das Vorliegen intraartikulärer freier Gelenkkörper beurteilt werden. Die Defektsituation des Coronoids lässt sich allerdings am besten in der Computertomographie beurteilen, weshalb sich auch diese Untersuchung additiv (idealerweise mit 3‑D-Rekonstruktion) anbietet.

## Operationsprinzip und -ziel

- Rezentrierung des Gelenks durch Wiederherstellung der knöchernen Gelenkkongruenz mittels autologen Beckenkammspans über einen medialen Zugang
- Simultane Rekonstruktion des medialen Kollateralbandes (AMCL) mittels autologem Gracilissehnengrafts

## Vorteile

- Wiederherstellung der Gelenkkongruenz zur Neutralisierung der PMRI und damit Vermeidung der Entwicklung einer Ellenbogenarthrose durch anhaltende Subluxationsstellung (Abb. 3)
- Möglichkeit der additiven medialen Bandplastik aufgrund des medialen Zugangsweges
- Keine zusätzliche Destabilisierung des Ellenbogens durch Radiuskopf- oder Olekranonspitzengraft [34, 35]
- Durch Entnahme am Beckenkamm variable Größenbestimmung und somit auch Möglichkeit zur Versorgung großer Coronoiddefekte

## Nachteile

- Entnahmemorbidität am Beckenkamm
- Nichtanatomische Rekonstruktion des Coronoids (Gelenkfläche ohne Knorpelüberzug mit bekannten potenziell negativen Konsequenzen)

## Indikationen

- Chronische PMRI nach Coronoidfraktur bei anteromedialem Facettendefekt
- Longitudinale Instabilität nach Coronoidbasisfraktur
- Akute PMRI bei nicht-rekonstruierbarer Coronoidfraktur

## Kontraindikationen

- Fortgeschrittene arthrotische Veränderungen des Ellenbogengelenks
- Fehlende Operabilität aufgrund allgemeiner (z. B. kardiovaskuläre Risikofaktoren) oder lokaler (z. B. Infektion) Komplikationen
- Schwangerschaft

## Patientenaufklärung

- Iatrogene Verletzung von neurovaskulären Strukturen mit sensiblen oder motorischen Ausfällen (v. a. N. ulnaris, auch N. radialis und N. medianus)
- Pseudarthrose bzw. sekundäre Dislokation des Beckenkammspans
- Entnahmemorbidität am Beckenkamm (Schmerzen, Narbe, Infekt) inklusiver möglicherweise eingeschränkter Mobilität für einige Tage
- Beckenfraktur (Os ilium)
- Nicht-vollständige Wiederherstellung der Gelenkkongruenz mit Entwicklung oder Zunahme einer Ellenbogenarthrose
- Postoperative Funktionseinschränkung (eingeschränkte Beweglichkeit, persistierender Schmerz)
- Lange Rekonvaleszenz inklusive Arbeitsunfähigkeit (zwischen 3 und 6 Monaten)

## Operationsvorbereitungen

- Narkoseuntersuchung (Überprüfung der Beweglichkeit, speziell im Vergleich zum präoperativen Zustand, einer posteromedialen und posterolateralen Rotationsinstabilität im Seitenvergleich und einer Luxationstendenz)
- Standardisiertes Time-Out zur Identifizierung des Patienten und Bestätigung der korrekten Seite und durchzuführenden Operation
- Rückenlagerung
- Blutsperre/Blutleere am Oberarm. Auslagerung des Armes auf einem steril bezogenen und höhenverstellbaren Beistelltisch
- Abdecken des abgewinkelt ausgelagerten ipsilateralen Beines sowie des Beckenkamms

## Instrumentarium

- Standardinstrumentarium für offene Ellenbogenoperationen
- Oszillierende Säge
- Hochfrequenzfräse
- K‑Drähte zur temporären Fixierung des Beckenkammspans
- Kanülierte Schrauben oder Osteosyntheseplatte zur endgültigen Retention
- 2er FiberLoop (Fa. Arthrex, Naples, Florida, USA)
- 4,5-mm-Bohrer
- 5,0 mm kanülierter Bohrer
- 2er Ösen-K-Draht
- Sehnenstripper (bei Sehnenentnahme am Kniegelenk) und Tenodeseschraube für die additive mediale Seitenbandplastik mittels Gracilissehne bei häufig assoziierter MCL-Insuffizienz

## Anästhesie und Lagerung

- Allgemeinnarkose (Larynxmaske/Intubation)
- Lokoregionale Analgesieverfahren (als Block oder idealerweise in Form eines Katheters) können ebenfalls zur Durchführung der Operation und zur postoperativen Schmerztherapie eingesetzt werden (auch additiv zur Vollnarkose)
- Rückenlagerung
- Blutsperre/Blutleere am Oberarm. Auslagerung des Armes auf einem steril bezogenen und höhenverstellbaren Beistelltisch
- Abdecken des abgewinkelt ausgelagerten ipsilateralen Beines sowie des Beckenkamms

## Operationstechnik

**Figure 4:**
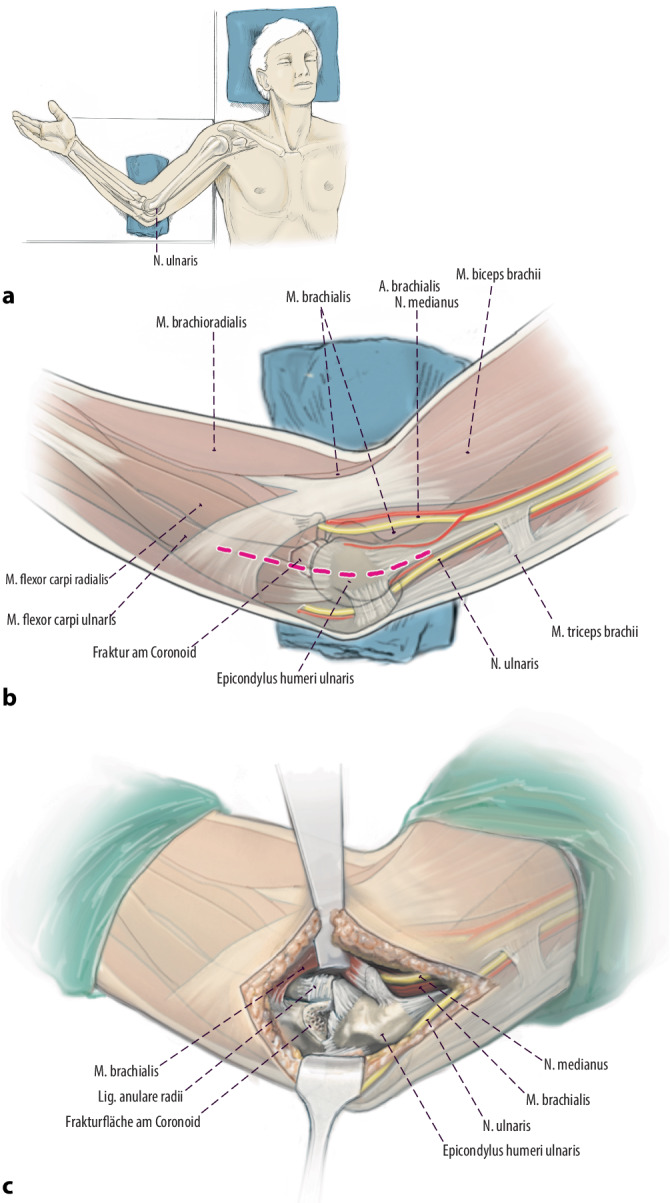

**Figure 5:**
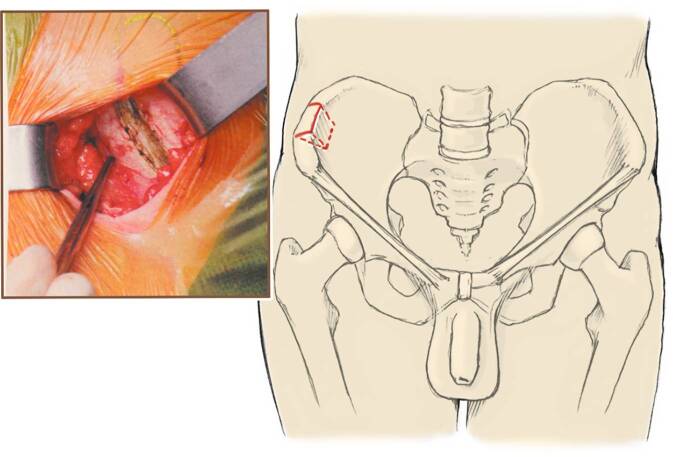

**Figure 6:**
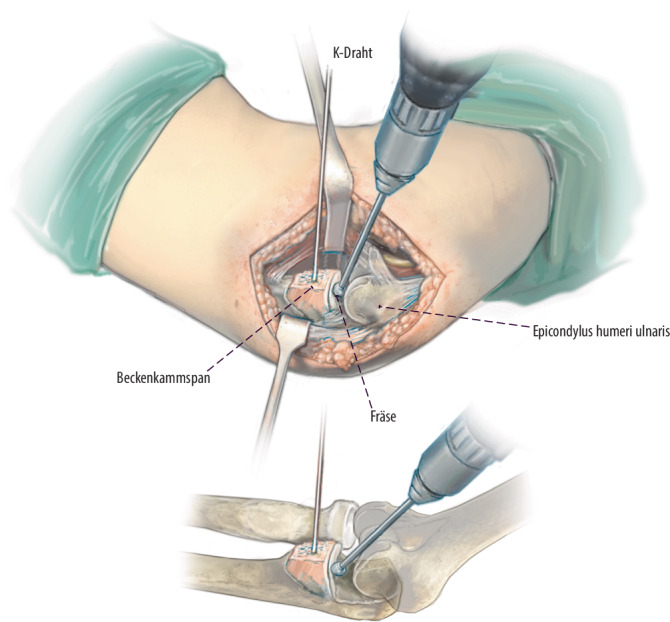

**Figure 7:**
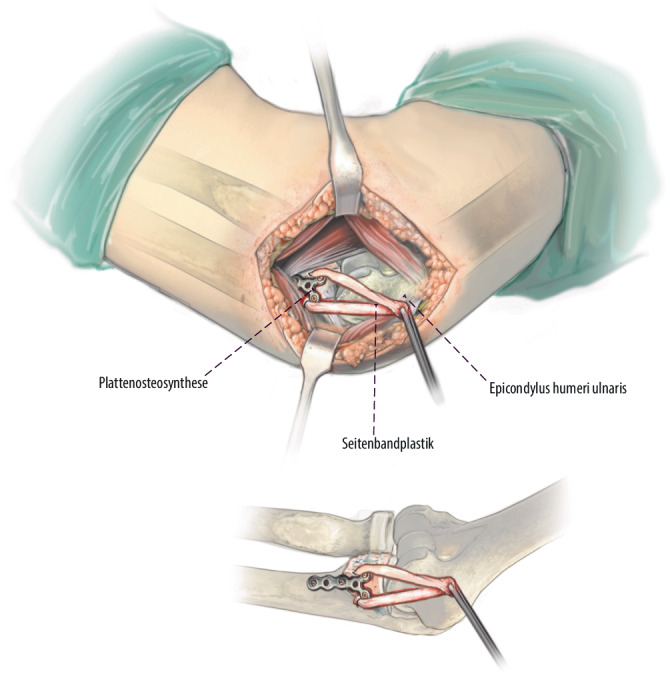

**Figure 8:**
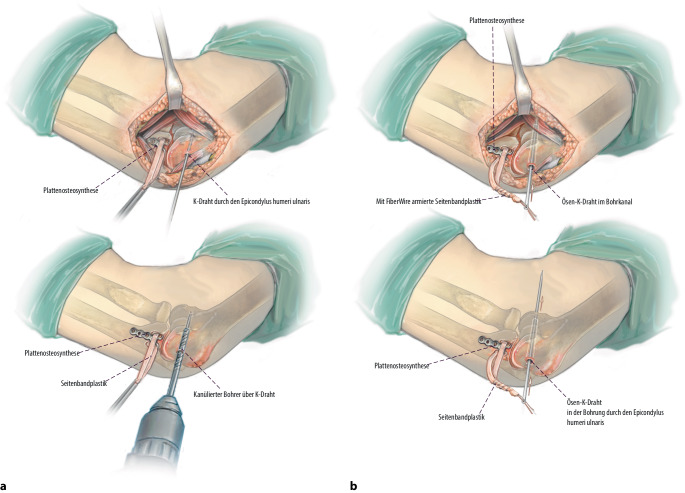

**Figure 9:**
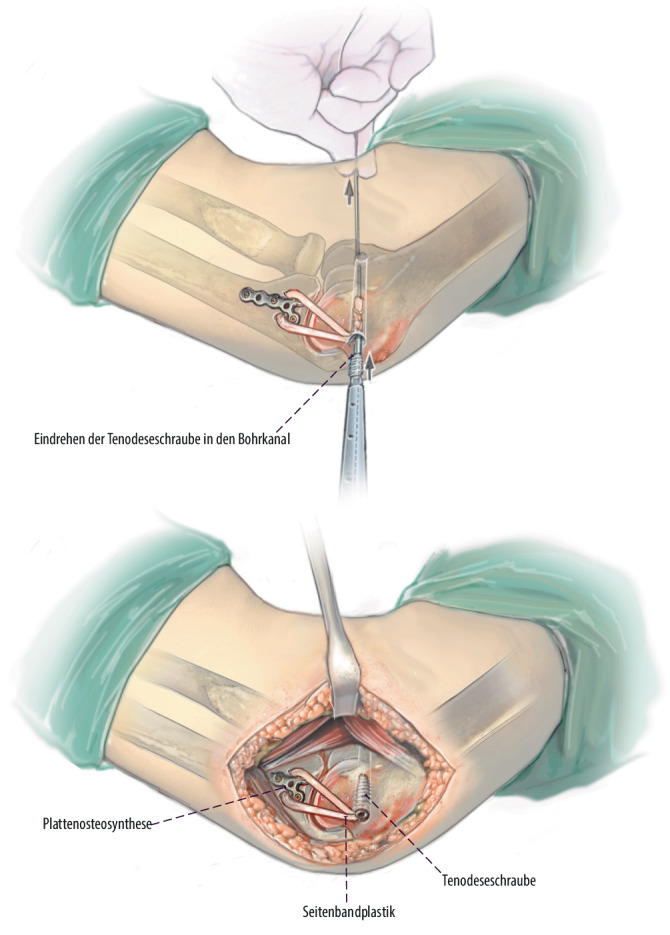

**Figure 10:**
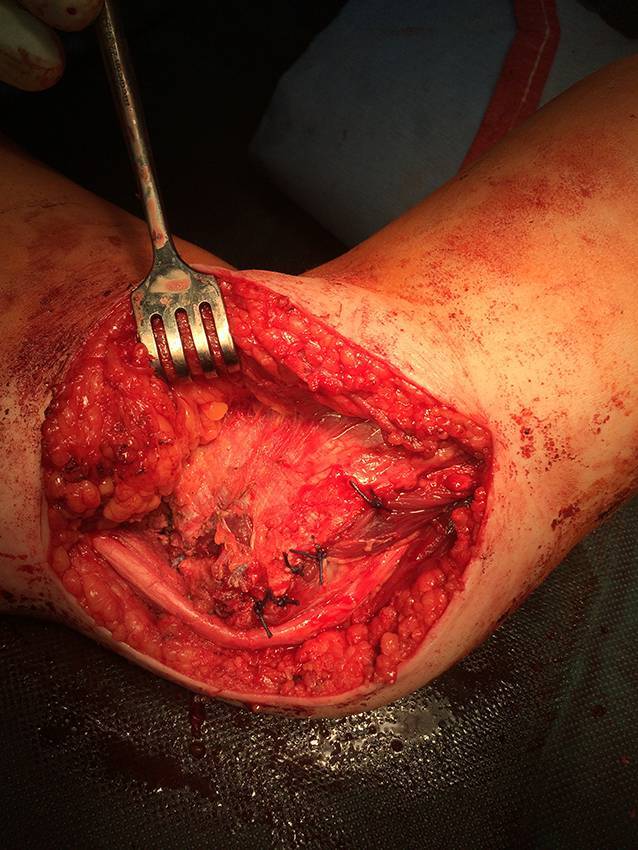

Abb. 4, 5, 6, 7, 8, 9, 10.

## Postoperative Behandlung

- Tragen einer intraoperativ angepassten Gipsschiene.
- Bei trockenen Wundverhältnissen (meist nach ca. 2 bis 4 Tagen) Wechsel auf Bewegungsorthese. Diese sollte für 6 Wochen Tag und Nacht getragen werden und nur für Physiotherapie bzw. Eigenübungen abgenommen werden
- Bei angelegter Orthese und aus ihr heraus tägliches Auftrainieren der Beweglichkeit in Flexion und Extension im Rahmen von Eigenübungen sowie der Krankengymnastik
- Verordnung eines CPM-Ellenbogenstuhls (z. B. Ellenbogenstuhl, Fa. Ormed, Freiburg, Deutschland) zur mehrfachen täglichen Anwendung zur Verbesserung der Beweglichkeit
- Verordnung von Lymphdrainage als abschwellende Maßnahme
- Postoperative Röntgenkontrolle in 2 Ebenen
- Regelmäßige Wundkontrollen sowie Verbandswechsel. Fadenzug am 10. bis 12. postoperativen Tag

## Fehler, Gefahren, Komplikationen

### Intraoperative Fehler und Gefahren

- Intraartikuläre Schraubenlage
- Fehlplatzierung des Knochenspans
- Fehlplatzierung der Bandplastik
- Fraktur der Spina iliaca anterior superior bei geminderter Knochenqualität bzw. zu weit ventraler Spanentnahme
- Fehlender Schutz bzw. Verletzung des N. ulnaris während der Operation

### Postoperative Komplikationen

- Allgemeine Komplikationen:
  - Allgemeine Komplikationen sind bei adäquatem Vorgehen sehr selten, a. e. Nachblutungen, postoperative Ellenbogensteife (Arthrofibrose), persistierende Schmerzen, postoperative Ellenbogensteife.
- Spezielle Komplikationen:
  - Pseudarthrose oder sekundäre Dislokation des Beckenkammspans
  - Zeitnahe Entwicklung einer Arthrose bei inkongruenter Gelenkflächenrekonstruktion
  - Iatrogene Schädigung des N. ulnaris
  - Extensions- oder Flexionskontraktur bei falscher Isometriebestimmung
  - Mediale Bandinsuffizienz bei Einheilungsstörungen oder Materialversagen
  - Einheilungsstörungen der rekonstruierten Flexorensehne
  - Entnahmemorbidität mit Stufenbildung am Beckenkamm und daraus resultierenden Einschränkungen (z. B. beim Tragen eines Gürtels)

## Ergebnisse

Die posteromediale Rotationsinstabilität (PMRI) ist eine seltene, aber inzwischen detailliert untersuchte Verletzung des Ellenbogens, welche akut und chronisch auftreten kann und sich durch eine relevante Fraktur der anteromedialen Facette des Processus coronoideus bedingt [5, 6, 18, 22, 36]. Dabei sind isolierte Frakturen selten, vielmehr werden die Verletzungen des Coronoids im Zuge einer Ellenbogenluxation bzw. komplexen Fraktursituation nicht ausreichend versorgt. Eine konservative Behandlung der Coronoidfraktur [23] oder eine alleinige Rekonstruktion des LUCL [37, 38] führt dabei oft zu schwerwiegenden Verläufen mit zeitnaher Entwicklung einer Ellenbogenarthrose bedingt durch die persistierende posteromediale Subluxationsstellung.

Der Schlüssel zur erfolgreichen Therapie dieser komplexen Pathologie liegt zum einen im Erkennen der Verletzungen und korrekten Einschätzung deren Ausmaßes (Begleitverletzungen!) und zum anderen im Verständnis von Anatomie und Funktionsweise des Coronoids und der ulnaren und radialen Seitenbänder [22]. In der Akutsituation kann eine Rekonstruktion der anteromedialen Facette mittels Osteosynthese oder Fadenankern mit – sofern notwendig – additiver medialer bzw. lateraler Bandrefixation die Entstehung einer chronischen PMRI vermeiden. Vor allem bei den chronischen Verläufen gestaltet sich die klinische Untersuchung schwierig, weil sich die Patienten oftmals ohne pathognomonische Symptome vorstellen. Aus diesem Grund kommt der Schnittbildgebung, idealerweise inklusive 3‑D-Rekonstruktion, eine besondere Bedeutung zu, in welcher die Fraktur nach O’Driscoll verlässlich interpretiert werden kann [39].

Zwischen April 2015 und November 2017 wurden von den Autoren 10 Patienten mit chronischer PMRI, bedingt durch einen Coronoiddefekt, in oben beschriebener Technik operiert. Das Durchschnittsalter der Patienten zum Zeitpunkt der Operation betrug 41,4 Jahre. Acht der 10 Patienten konnten 86,3 Wochen [Range: 53–137 Wochen] postoperativ klinisch-radiologisch nachuntersucht werden. Ein Patient musste im Verlauf aufgrund einer posttraumatischen Arthrose auf eine Ellenbogenprothese konvertiert werden und wurde deshalb aus dem Patientenkollektiv ausgeschlossen.

Neben einer Röntgenbildgebung des Ellenbogens in 2 Ebenen erfolgte eine klinische Untersuchung des Ellenbogens sowie die Erhebung des „Subjective Elbow Value“ (SEV), des aktuellen Schmerzniveaus anhand der visuellen Analogskala, des „Disabilities of the Arm, Shoulder and Hand“(DASH)-Scores [40] sowie des „Mayo Elbow Performance Score“ (MEPS) [41].

Der Bewegungsumfang hat sich durch die Operation im Mittel von 108° auf 136° (Flexion) bzw. von 41° auf 16° (Extension) verbessert. Der durchschnittliche Grad der Supination verbesserte sich von 61° auf 82°, der der Pronation von 54° auf 60°. Der SEV konnte im Vergleich zum präoperativen Zustand von 44 % auf 81 % verbessert werden. Der DASH-Score fiel von 50 (9–91) auf 26 (0–57) Punkte und dokumentierte damit eine positive Entwicklung. Der Mittelwert des MEPS konnte von 39 (10–60) auf 81 (60–100) Punkte gesteigert werden. Demgegenüber fiel das angegebene Schmerzlevel der Patienten auf der visuellen Analogskala in Ruhe bzw. unter Belastung im Vergleich zum Zeitpunkt vor der Operation von 6,25 (3–9) bzw. 8 (6–10) auf 1,1 (0–2,5) bzw. 3,6 (0–6). Bei 7 Patienten war zusätzliche eine LUCL-Bandplastik notwendig, welche im eigenen Vorgehen in allen Fällen sekundär durchgeführt wurde. In der klinischen Nachuntersuchung konnte bei einem dieser Patienten eine leichte posterolaterale Rotationsinstabilität im Pinzettengriff festgestellt werden. Keiner der nachuntersuchten Patienten erlitt eine erneute Ellenbogenluxation. Aufgrund einer Ellenbogensteife musste bei einem Patienten eine arthroskopische Arthrolyse zur Verbesserung der Beweglichkeit durchgeführt werden. Niemand aus dem Kollektiv wies postoperativ eine ulnare Instabilität auf (negativer Valgusstresstest, schmerzfreier Varusstresstest, negatives Milking-Manöver). Ein Patient klagte über eine Hypästhesie in den Fingern im Versorgungsgebiet des N. ulnaris, 2 weitere Patienten über eine dauerhafte Hypästhesie im Bereich der Narbe. Wie oben beschrieben, entwickelte sich bei 1 Patienten eine fortgeschrittene Ellenbogenarthrose, weshalb die Konversion auf eine Ellenbogentotalendoprothese notwendig war. In den radiologischen Nachuntersuchungen zeigten sich bei allen 7 Patienten regelrechte Gelenkstellungen ohne vorliegende Subluxationsstellung, allerdings wiesen 3 Patienten Zeichen einer beginnenden posttraumatischen Arthrose auf.

Klinische Ergebnisse von Patienten, deren Verletzung initial unterschätzt und im Verlauf wegen chronischer PMRI mittels Coronoidaufbauplastik therapiert wurde, sind in der Literatur selten und beschränken sich auf kleine Fallserien oder Case Reports mit mehr oder weniger langen Follow-up-Zeiträumen [25, 26, 29, 42–44]. Die Ergebnisse einer größeren Fallserie (*n* = 44) mit einem durchschnittlichen Follow-up von 5,8 Jahren (*n* = 26) wurden während des Jahreskongresses der DVSE 2018 in Regensburg präsentiert [45], wobei die Coronoidrekonstruktion mittels Beckenkammspan nicht wie in diesem Beitrag von medial, sondern über einen anterioren Zugang durchgeführt wurde [46, 47].

In der Literatur ist der Coronoidaufbau nicht nur mittels autologen Beckenkammspans [25, 27, 42] beschrieben, sondern wird auch mit allogenem oder autologem Radiuskopfgraft [9, 29, 44, 48, 49], einem costochondralen Graft [50] oder der Olekranonspitze [26] dargestellt. Die Vorteile von Radiuskopf- und Olekranonspitzengraft liegen in der geringeren Entnahmemorbidität und der Möglichkeit der Wiederherstellung einer knorpligen Gelenkfläche bei hoher Passgenauigkeit [44, 51]. Der Radiuskopf wird v. a. dann verwendet, wenn aufgrund einer Defektsituation eine prothetische Versorgung notwendig ist. Bei chronischen Defekten erscheint eine Entnahme des intakten Radiuskopfes obsolet, da dies zu einer zusätzlichen Destabilisierung des bereits kompromittierten Ellenbogens führt [34, 35, 52]. Die Olekranonspitze kann zwar theoretisch sowohl in der akuten und chronischen Situation verwendet werden, allerdings ist dieses Graft aus eigener Erfahrung bei den chronischen Fällen oft zu klein, um das Coronoid adäquat zu rekonstruieren. Allen genannten Studien ist gemeinsam, dass der präoperative Zustand verbessert werden konnte, sich in den Folgeuntersuchungen – wie auch in unserer Studie – allerdings radiologisch degenerative Veränderungen zeigten, die sich trotz der Re-Zentrierung des ulnohumeralen Gelenks nicht vollständig aufhalten lassen und am ehesten durch den Unfallmechanismus und die aus der osteoligamentären Verletzung resultierenden Subluxationsstellung verursacht werden.

Zusammengefasst stellt die Rekonstruktion des Coronoids mittels Beckenkammspan und additiver Bandplastik bei korrekter Durchführung eine adäquate Therapie zur Wiederherstellung der Kongruenz und Rezentrierung des Ellenbogengelenks dar, mit der präoperative Beschwerden reduziert werden können und eine Arthroseentwicklung zumindest ausgebremst werden kann. Vergleichsstudien und Langzeitergebnisse sind allerdings notwendig, um den Coronoidaufbau noch besser einordnen zu können. Idealerweise werden Verletzungen bzw. eine Beteiligung des Coronoids in komplexen Fraktursituationen oder Ellenbogenluxationen initial erkannt und korrekt behandelt, so dass es gar nicht erst zur Ausbildung einer chronischen PMRI kommen kann.

In der von uns durchgeführten Studie ließen sich mit der beschriebenen Technik objektive wie subjektive Verbesserungen erzielen. Kein Patient zeigte eine erneute Reluxation und die Patienten waren mit dem Ergebnis des Behandlungsverlaufs zufrieden. Limitationen der Studie sind neben dem retrospektiven Charakter auch die geringe Fallzahl.
